# Improving preparedness for mass casualty incidents in hospitals: insights from a large-scale simulation exercise with geotracking and validated questionnaires

**DOI:** 10.1186/s12873-026-01527-6

**Published:** 2026-03-09

**Authors:** Maik von der Forst, Hanne Schaefer, Stefan Mohr, Hannes G. Kenngott, Elyes Farjallah, Matthias Huck, Anke S. Baetzner, Marie Ottilie Frenkel, Markus Ries, Martin Loos, Christoph W. Michalski, Christine Leowardi, Markus Weigand, Erik Popp, Gabriel A. Salg

**Affiliations:** 1 Medical Faculty Heidelberg, Department of Anesthesiology, Heidelberg University, Im Neuenheimer Feld 420, 69120 Heidelberg, Germany; 2 Medical Faculty Heidelberg, Medical Crisis and Disaster Management Unit, Heidelberg University Hospital, Im Neuenheimer Feld 420, 69120 Heidelberg, Germany; 3 Medical Faculty Heidelberg, Department of General-, Visceral- and Transplantation Surgery, Heidelberg University, Im Neuenheimer Feld 420, 69120 Heidelberg, Germany; 4 Institute of Sports and Sports Sciences, Heidelberg University, Heidelberg, Germany; 5 Medical Faculty Mannheim, Department of Public Mental Health, Central Institute of Mental Health, Heidelberg University, Mannheim, Germany; 6 Faculty Health, Medical & Life Sciences, Psychology in Healthcare, Furtwangen University, Freiburg, Germany; 7 Medical Faculty Heidelberg, Pediatric Neurology and Metabolic Medicine, Center for Pediatrics and Adolescent Medicine, Heidelberg University, Im Neuenheimer Feld 430, 69120 Heidelberg, Germany

**Keywords:** Mass casualty incident, Disaster medicine, Hospital disaster planning, Critical infrastructure, Leadership, Communication, Incident command

## Abstract

**Background:**

Mass casualty incidents (MCIs) rapidly exceed routine hospital capacity. Full-scale exercises are essential for preparedness, but systematic, multidimensional evaluations remain scarce. This study aimed to evaluate overall team performance in triage accuracy, workflow, and individual workload throughout a MCI exercise.

**Methods:**

In a prospective observational study at Heidelberg University Hospital (Germany), healthcare professionals managed 91 simulated casualties using a two-stage triage process. Patients and staff carried location tags enabling continuous spatiotemporal tracking. Objective outcomes included triage accuracy, triage duration, patient flow, and staff–patient contact frequency. Subjective workload and teamwork were assessed using the NASA Task Load Index (NASA-TLX) and the Team Emergency Assessment Measure (TEAM), respectively.

**Results:**

Overall triage accuracy was 75.4%. Undertriage occurred in 11.6% of category I and 10.1% of category II cases; overtriage was infrequent (2.9%). Mean triage times differed significantly by category: ‘red’ 59 ± 25 s, ‘yellow’ 173 ± 74 s, ‘green’ 205 ± 100 s (*p* < 0.0001). Geotracking demonstrated consistent patient flow without detectable bottlenecks and a mean of 7.1 ± 5.7 patient contacts per staff member. NASA-TLX scores indicated high temporal demand but low frustration with an overall workload of 66.7 ± 16; specialists and staff with greater professional experience reported significantly lower perceived workload (*p* < 0.05). TEAM ratings were homogeneously good across all participants (79.8%).

**Conclusions:**

This study provides reproducible benchmark data on simulated hospital MCI response. The integration of geotracking with subjective measures, enables a comprehensive evaluation of hospital disaster preparedness. Moreover, the ability to compare different exercises and collect reliable longitudinal data may further enhance hospital disaster response.

**Supplementary Information:**

The online version contains supplementary material available at 10.1186/s12873-026-01527-6.

## Introduction

Mass casualty incidents (MCIs) challenge healthcare systems [1–3]. A sudden influx of numerous injured patients can quickly exceed normal operational capacities of hospitals. Past events suggest that regular practice leads to improved patient care in real-life situations [4–8]. Exercises designed to train for MCIs have been carried out for many decades [9, 10]. Full-scale simulation exercises are recognized as a vital component in enhancing hospital preparedness for such events [11–13]. Such exercises allow healthcare professionals to practice and refine their response protocols in a controlled, yet realistic environment. Thereby potential weaknesses can be identified and addressed before an actual event occurs [14]. Evidence highlights the value of simulation exercises in improving various aspects of hospital preparedness, including the coordination and decision-making abilities of medical teams under stress, as well as the overall effectiveness of emergency response protocols [15–19]. Previous research has shown that regular simulation training can enhance the situational awareness and communication skills of healthcare providers, leading to more efficient and effective patient care during emergencies [11, 12, 20]. It seems imperative to maximize the output from each simulation exercise. Structured evaluation and collection of metric data during MCI training allows for the generation of evidence that can inform long-term improvements in emergency response strategies and healthcare system resilience [21]. However, the organization and execution of full-scale MCI simulations are fraught with difficulties [22]. Organizational, budgetary, workload and resource constraints cause infrequent execution [22–24].

Therefore, there is a notable lack of comprehensive studies that integrate both objective and subjective data to provide a holistic view of hospital performance during MCI simulations. To address this gap, this study conducted a full-scale MCI simulation designed to obtain a multidimensional evaluation of the operational processes. By integrating objective and subjective assessment methods, the present study aimed to achieve a comprehensive analysis of interdisciplinary treatment teams’ performance during MCIs.

## Methods

### Study design

This is a prospective observational study approved by the ethics committee of the medical faculty of the University of Heidelberg (S-556/2023) and conducted in accordance with the current version of the Declaration of Helsinki. Clinical trial number: not applicable. Patients and/or the public were not involved in the design, conduct, reporting or dissemination plans of this research. Employees of Heidelberg University Hospital that participated in the hospital MCI simulation exercise represent the study population. After written informed consent to participate in the study was provided, each participant was registered anonymously using a unique code. In total 75 participants were additionally equipped with a location tag, that was registered under the same code. Immediately after finishing the simulation exercise each participant was asked to answer two standardized questionnaires as well as complete demographic and baseline data registered to the unique code. Data from all modalities were merged using the unique code, which was immediately deleted afterwards to prohibit de-anonymization. Participants received no additional training prior to the MCI simulation exercise and were aware only on the date of the full-scale exercise. Missing data were not imputed. STROBE criteria were adhered to in design, conduct, and reporting of this observational study [25].

**Fig. 1:**
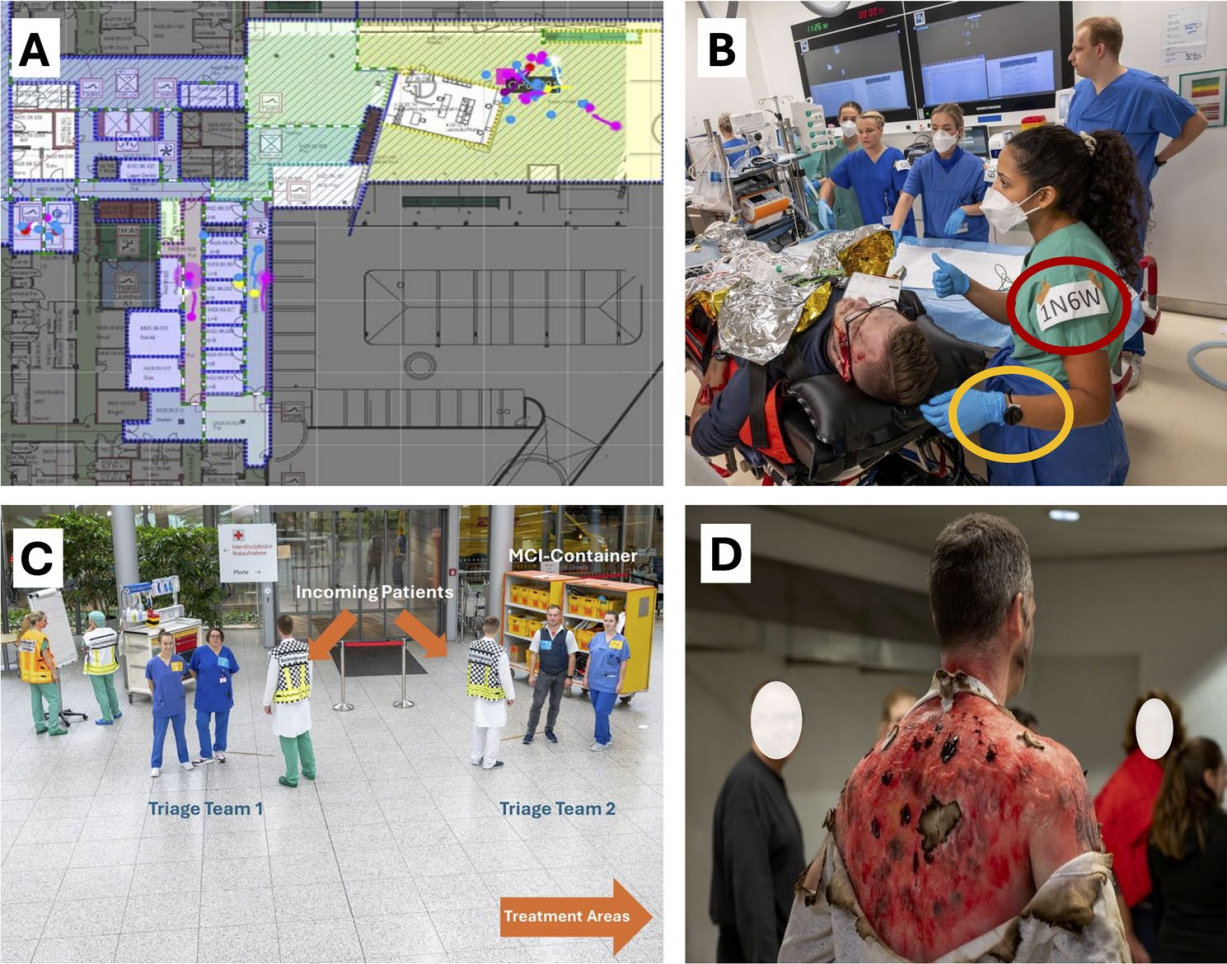
Impressions of the full-scale MCI exercise. **A** Geotracking of patients and participants during the simulation exercise in the different areas. For further insights see also Supplemental Video 1. **B** Patient and medical team in the category I (‘red’) treatment area (red circle: unique code for study participation, yellow circle: tags for geo-tracking). **C** Set-up of the algorithm-based triage point. **D** Simulation patient with realistic makeup injuries

### Full-scale MCI simulation exercise

The exercise was conducted at Heidelberg University Hospital (Germany) on a Saturday morning in November and involved 91 simulated casualties (Fig. 1). The clinic regularly conducts theoretical training and continuing education courses on dealing with MCIs. Smaller exercises in various areas take place about once a year, while large full-scale exercises are planned every five years. Participation in the exercises is usually voluntary but may be mandatory.

The scenario for this exercise involved an accidental explosion in the boiler room of a public building. The accident occurred during repair work on the heating system in the basement. At the same time, a training course was taking place in the building. The explosion injured employees of the heating company and course participants. The injuries were determined as realistically as possible in accordance with the scenario and the planned distribution was: 30% category I patients, 30% category II patients, and 40% category III patients. Injuries included: impalement of the thorax, open abdomen after a fall, sternum fracture, multiple rib fractures, hemopneumothorax, amputations of extremities, burns, inhalation trauma, open fractures, lacerations, paraplegia after spinal trauma, traumatic brain injury, eye injuries, blast trauma with deafness, knee injuries, sprains, abrasions, and other soft tissue injuries. Approximately 20% of Category I patients had abdominal injuries, 20% had thoracic injuries, 20% had amputations, and 20% had burns, often in combination with other polytrauma injuries. The remainder had severe traumatic brain injuries, lacerations, or similar injuries.

The patients were simulated by a professional team of patient actors who have extensive experience and training in both makeup and the simulation of specific injuries. The presentation of injuries followed pre-defined case vignettes (Fig. 1). The actors were assigned appropriate roles that included both the injury pattern and the vital signs to be simulated. These were presented visually or through acting (e.g., breathing rate) whenever possible; otherwise, they were communicated to the medical teams after realistic assessment (e.g., blood pressure measurement). The actors also had a certain dynamic in their roles, so that, for example, there was a deterioration if certain measures were not taken.

### Triage process and procedures during the exercise

The simulation exercise began with a mass casualty incident alarm at the hospital at 10:21 a.m. and ended with the treatment of the last patient at 1:48 p.m., meaning that the exercise lasted a total of 3 h and 27 min. At the start of the exercise, the participating employees were not yet in the hospital, except for the regular staff. This means that travel times, changing times, staff registration, team formation, etc. were also simulated and taken into account. The first patient arrived at the hospital after 40 min. Thereafter, patients arrived at irregular intervals, sometimes in waves, in line with a realistic scenario.

Upon arrival of patients the triage at the hospital was performed in a two-stage process. At a first stage patients were classified at “first look” into the three categories by skilled emergency room nurses trained in everyday assessment of patients. This first triage point was set up at the driveway to the trauma center’s emergency department. The scope was directing vehicles with category I and II (‘red’ and ‘yellow’) patients to the emergency department entry and vehicles with category III (‘green’) patients to the ground level entrance. This simple distinction had a tactical value, namely that the minor injuries did not block the important underground garage access to the operating room and intensive care units to avoid a bottleneck in the care of critical patients.

At a second stage two identically equipped triage points were set up at each respective entrance (Fig. 1) and a more sophisticated triage was performed by a tandem of general/trauma surgeon and anesthesiologist. A proprietary triage algorithm was used according to the hospital`s alarm and operation plan, classifying the patients in the known categories: Immediate life-threatening condition (category I, ‘red’), serious injuries, but no immediate needs (category II, ‘yellow’) or minor injuries such as abrasions and smaller lacerations (category III, ‘green’) (Supplemental Fig. 1). The algorithm is largely identical to the Berlin algorithm for in-hospital triage, which has been validated and published several times. Only the order of the items for each category has been adapted to the cABCDE scheme, and life-saving emergency measures have been added. In addition, the items “peri-arrest situation” and “airway obstruction or intubated patient” have been included as criteria for category I [26, 27]. After triage, patients were assigned to treatment teams of at least three healthcare professionals (including at least one physician). The teams were formed spontaneously upon arrival and registration at the trauma center. The teams performed initial treatment or ordered further diagnostic measures (ultrasound, x-ray, computed tomography) or emergency surgery according to their knowledge and skills. The simulation was stopped before transfer to a respective ward or after arrival at an assigned operating theatre. There has been additional staff responsible for operating theatre coordination, surgical assistance and anesthesiologic assistance. Once, the treatment simulation for one patient was completed, the teams were ready for their next case assignment. 

### Real-time location system (RTLS)

Locators and tags were provided and set up by Favendo GmbH (Bamberg, Germany) to achieve a maximum resolution <1m. Triage areas as well as category I and category II treatment areas and transport areas (Fig. 1) were equipped with locators. Participants and patients were randomly equipped with tags (Fig. 1). Based on RTLS, triage time was measured and defined as the time from arrival at the second triage area until transport out of the triage area. Further, triage accuracy as well as potential over- and undertriage was analyzed. Data preparation was performed using a customized python script (Python Software Foundation, Python Language Reference, Version 3.10.). For visualization, the quupa player (Quuppa Data Player, QDP) was used for each respective floor and respective tags were annotated with regard to the pre-defined triage category (‘red’/’yellow’/’green’) or respective professional group of the study participants. A video illustrating real-time tracking of the emergency department level (Category I and Category II patients) is provided in the supplement to this manuscript (Supplemental Video 1). The spatiotemporal resolution rate corresponded to a sampling process every 500 ms. Patient contact was defined as the proximity of an employee tracker and a patient tracker of < 1 m for a period of at least 1 s. 

### Demographic and baseline data

Participants were asked to provide the following data linked to their unique code: age (in years), professional group (‘physician’/’nurse’/’nurse trainee’/’other’), professional experience (in years). Physicians were further asked whether they have completed their respective specialist training. Gender was assessed by self-report using the German term ‘Geschlecht’, with response options ‘male’, ‘female’, and ‘diverse’. 

### National Aeronautics and Space Administration-Task Load Index (NASA-TLX)

The NASA-TLX was completed immediately after the exercise. It is a subjective, multidimensional assessment tool for recording perceived workload with six subscales (mental demands, physical demands, time demands, personal performance, effort and frustration), each of which is rated on a scale of 1 to 20, high numbers indicating a high workload. The NASA-TLX is an established tool to measure perceived workload when performing a task [28–30]. 

### Team Emergency Assessment Measure (TEAM) scale

The Team Emergency Assessment Measure (TEAM) is a subjective, multidimensional rating instrument designed to evaluate non-technical performance of emergency teams. It focuses on key aspects such as leadership, communication, coordination, and task management. The TEAM comprises 11 items rated on a five-point Likert scale, ranging from ‘0’ (Never/hardly ever) to ‘4’ (Always/nearly always). These items assess three key dimensions: ‘Leadership’ (2 items), ‘Teamwork’ (7 items), which includes aspects of situational awareness, and ‘Task Management’ (2 items) and altogether non-technical team performance. The TEAM total score was calculated as a sum of the 11 eleven section items with a maximum score of 44 as reported previously [31–33]. Additionally, a 12th item provides a global performance rating on a scale from 1 to 10 [31]. The German version of TEAM was used for this study [34]. Traditionally, rating is performed for the whole team by one observer [31, 32]. Here, the TEAM rating was performed immediately after the exercise by the participants themselves. 

### Statistical analysis

Data analysis and statistical testing was done using GraphPad Prism version 10.6.0 for Windows (GraphPad Software, San Diego, California, USA). Missing data was considered missing completely at random and no imputation was performed. Normality of the data was assessed using both the Shapiro–Wilk test and the Kolmogorov–Smirnov test with Lilliefors adaptation. Data that followed normal distribution were tested for statistically significant differences using the two-tailed t-test for comparison of two groups and the one-way ANOVA for comparisons involving three and more groups. An F-Test was conducted and if necessary, Welch’s correction was applied. Data that did not follow normal distribution were tested using the non-parametric two-tailed Mann-Whitney test for comparisons of two groups and the non-parametric Kruskal-Wallis test for comparisons of three and more groups. A p-value < 0.05 was considered statistically significant. Effect sizes were calculated using Hedge’s g, which provides a bias-corrected standardized mean difference suitable for small sample sizes. 95%-confidence intervals (95% CI) for Hedge’s g were computed to provide an estimate of the precision of effect size. As abbreviations for p-value summary *, **, ***, **** is used to indicate p-values < 0.05, < 0.01, < 0.001 and < 0.0001, respectively. Non-significance is indicated by the abbreviation *ns*.

## Results

Table 1 shows the characteristics of the exercise participants broken down by occupational group.

**Table 1:** Demographics of MCI Exercise Participants

Professional groups	*n*	ratio [%]			
Physicians	46	46.9			
Resident	*27*	*58.7*			
Specialist	*19*	*41.3*			
Nursing staff	41	41.8			
Nursing trainees	6	6.1			
Others	5	5.1			
**Gender**	*n (female)*	*ratio [%]*	*n (male)*	*ratio [%]*	*n (diverse)*
All participants *	52	53.1	46	46.9	0
Physicians	16	34.8	30	65.2	0
Nursing staff *	30	75.0	10	25	0
Nursing trainees	4	66.7	2	33.3	0
Others	2	40.0	3	60	0
**Age [y]**	*mean*	*SD*	*median*	*range*	
All participants *	35.7	10.5	32.0	20–66	
Physicians	34.4	6.1	33.0	26–49	
Nursing staff	37.5	12.2	31.0	21–66	
Nursing trainees	22.0	2.2	21.0	20–25	
Others	51.0	10.4	52.0	32–61	
**Professional experience [y]**	*mean*	*SD*	*median*	*range*	
All participants **	10.9	11.0	5.0	0–44	
Physicians	7.2	6.0	5.0	0–22	
Nursing staff	14.9	13.3	9.0	0.5–44	
Nursing trainees	2.3	1.1	3.0	0–3	
Others	24.8	10.5	29.0	7–34	

*completeness of response: 98.0%

**completeness of response: 97.0%

The average age of the participants was 35.7 years and was similar across medical and nursing staff (Mann-Whitney test, *p* = 0.346). Nevertheless, nursing trainees were significantly younger than nursing staff (excl. trainees) (*p* < 0.0001) and residents younger than specialists (*p* < 0.0001). Overall, the nursing staff had a median of 7 years of professional experience and the physicians a median of 5 years, this difference was not significant (*p* = 0.088, Table 1). A similar number of physicians were recruited as nursing staff. Taken together 53.1% of participants were female and the majority of these were nurses (73.3%).

### Triage accuracy and duration

**Table 2:** Results of Patient Triage and Patient Contacts per Participant

Triage accuracy, triage point 1, “first look”	*n* = 69	ratio [%]	
Overall accuracy	53	76.8	
Undertriage	14	20.3	
Missed Cat. I, ‘red’	7	10.1	
Missed Cat. II, ‘yellow’	7	10.1	
Overtriage	2	2.9	
False Cat. I, ‘red’	2	2.9	
**Triage accuracy**,** triage point 2**,** “algorithm-based"**	*n* = 69	*ratio [%]*	
Overall accuracy	52	75.4	
Undertriage	15	21.7	
Missed Cat. I, ‘red’	8	11.6	
Missed Cat. II, ‘yellow’	7	10.1	
Overtriage	2	2.9	
False Cat. I, ‘red’	2	2.9	
**Number of patient contacts per person**	*n*	*mean*	*SD*
All tracked staff	75	7.1	5.7
Tracked physicians	41	7.9	6.1
Tracked nursing staff	28	6.5	5.6
Tracked nursing trainees	4	6.3	1.3
**Patient contacts: Mann-Whitney test**	*P*	*P summary*	
Physicians vs. nursing staff (incl. trainees)	0.1310	ns	
Resident vs. specialist	0.9841	ns	
Nursing staff vs. trainees	0.2800	ns	
Female vs. male	0.7346	ns	
Prior participation	0.6787	ns	
Young vs. old	0.6902	ns	
Professional experience	0.2997	ns	

ns: not significant

Real-time location tracking of 69 patients was achieved. “First look” triage, led by two emergency department nurses, was conducted at triage point 1 in front of the hospital. The accuracy according to predefined case vignettes was 76.8%. An “algorithm based” second triage at triage point 2 (Fig. 1) was led by physicians with an overall triage accuracy of 75.4%. At triage point 2, 15 patients (21.7%) were undertriaged (eight ‘red’ vignettes triaged as ‘yellow’, seven ‘yellow’ vignettes triaged as ‘green’; Table 2). The ‘red’ vignettes triaged as yellow show no specific commonality, two patients had a leading abdominal trauma, four serious burn injuries, two major amputations with hemorrhagic shock and one thorax trauma with respiratory distress. Instead, going into further detail for undertriaged yellow vignettes reveals that 85.7% of patients falsely triaged as ‘green’ were blast trauma patients, which had deafness and bleeding from the ear as major symptoms. In total, only 2 patients (2.4%) were overtriaged (two ‘yellow’ vignettes triaged as ‘red’; Table 2).

Altogether *n* = 536 patient contacts were logged during the exercise. A comparison of the number of patient contacts between the different exercise participants revealed a homogeneous distribution. Nurses had approximately 6.5 patient contacts, while physicians had approximately 7.9 patient contacts; however, this difference was not significant. On average, participants had 7.1 patient contacts. Furthermore, there were no significant differences in patient contacts within the professional groups in terms of age, gender, or professional experience (Table 2). Data from one patient were lost due to technical failure of the location tag.

**Fig. 2:**
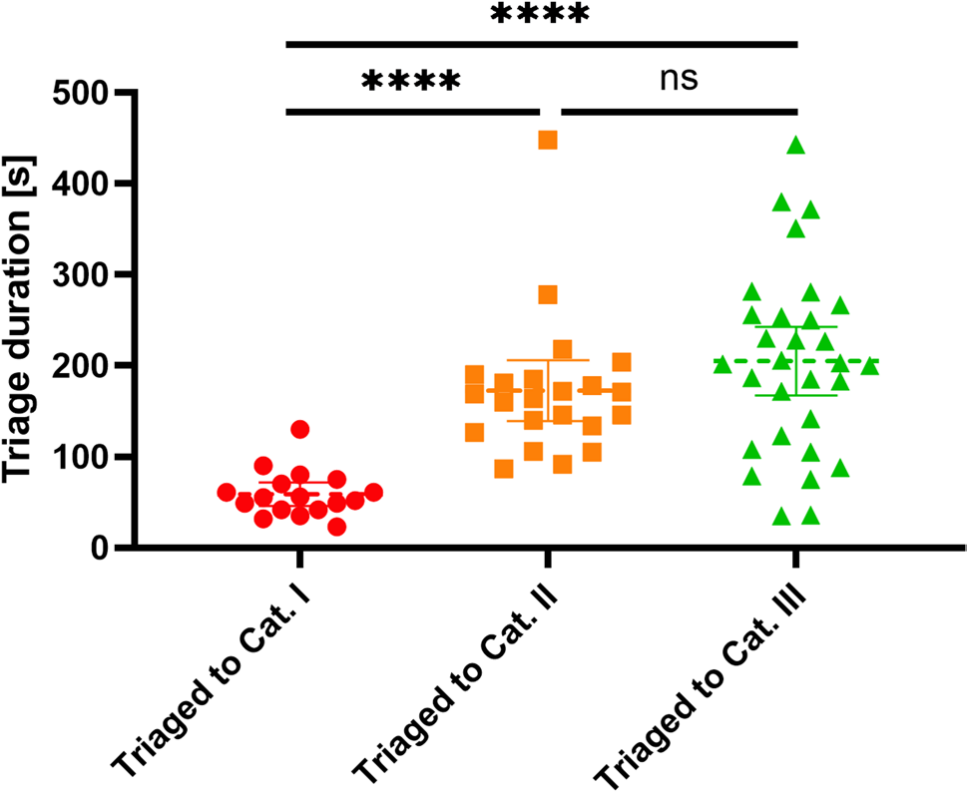
Comparison of Triage times by assigned Category. The figure shows the time required to examine a patient according to the assigned category. The times were automatically recorded based on the length of stay at the triage point using tracking data. Mann-Whitney test, **** *p* < 0.0001, ns: not significant

The mean triage time was 159 ± 98s overall. Analysis based on the actual triage category assignment revealed a mean triage time of 59 ± 25s for patients triaged as ‘red’, 173 ± 74s for patients triaged as ‘yellow’ and 205 ± 100s for patients triaged as ‘green’. The triage of ‘red’ patients was therefore significantly faster (*p* < 0.0001) compared to both other categories (Fig. 2). In comparison with the correctly triaged Category I patients, the triage time for undertriaged patients was significantly longer at a mean triage time of 192 ± 104s (*p* < 0.0001).

### Workload and team performance perception

The NASA-TLX had an overall high response rate of 99%. The emergency medical teams consisted of different professional groups and usually contained at least one nurse and one physician, further also nursing trainees were part of the teams (Supplemental Table 1). Regarding all participants, the items’ performance (13.9) and temporal demand (13.0) in the mean were rated highest, while physical demand (9.2) and frustration (7.2) where perceived less challenging (Supplemental Table 1). The individual items of the NASA-TLX show that physicians overall reported significantly larger *“effort”* than nursing staff (*p* < 0.05; g_Hedge_ = 0.50, [0.072–0.931]). Participants with less professional experience also reported significantly greater *“effort”* (*p* < 0.05; g_Hedge_ = 0.38, [-0.02-0.79]), higher *“physical demand”* (*p* < 0.05, g_Hedge_ = 0.41, [0.01–0.82]), and even higher *“frustration”* (*p* < 0.01, g_Hedge_ = 0.64, [0.29–1.05]) (Supplemental Table 1). Although “*frustration*” was rated relatively low overall as an item, this is consistent with the fact that *“frustration”* was higher among younger participants and resident physicians than among specialists. The same applies to nursing trainees, although the difference was not significant (*p* = 0.0511; Supplemental Table 1).

**Fig. 3:**
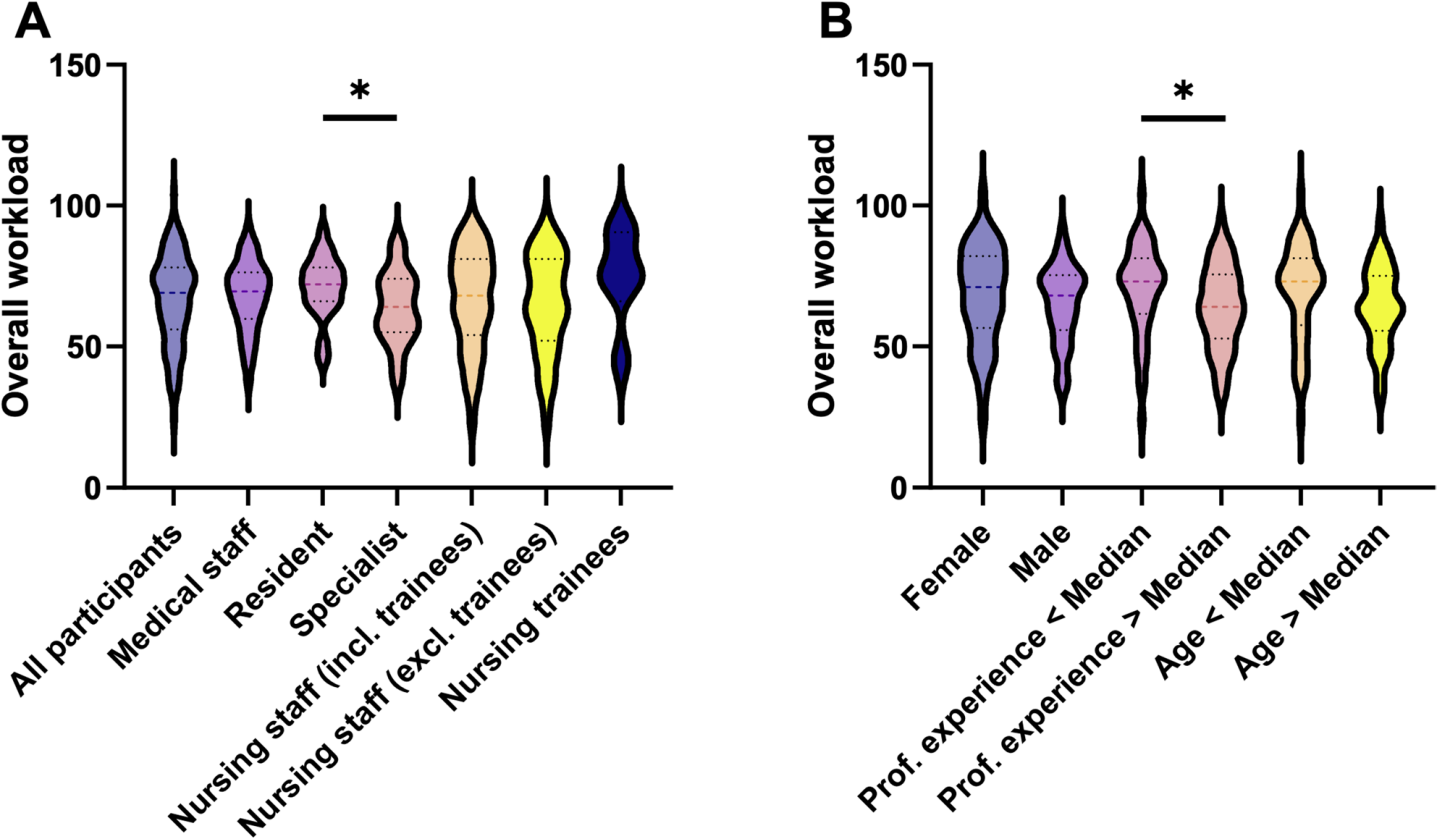
Overall workload according to NASA-TLX based on various participant characteristics. The overall workload is shown according to the “Raw TLX” by adding up the individual items. **A** shows the comparison based on the different professional groups and levels of training, while **B** compares the overall workload based on gender, age, and professional experience. Unpaired t-test, if necessary with Welch`s correction. * *p* < 0.05

A comparison of the overall workload shows that the perceived level is very similar across the different occupational groups. Nevertheless, specialist status of physicians is associated with a significantly lower overall workload compared to the residents (63.7 ± 13.0 vs. 71.1 ± 11.0, *p* < 0.05, g_Hedge_ = 0.62, [0.02–1.22]; Fig. 3) and nursing trainees showed a tendency toward a higher overall workload (75.2 ± 16.9), although there was no statistically significant difference (*n* = 6). Once professional experience is considered independently of the occupational group, the more experienced half of the participants perceives a significantly lower overall workload (63.8 ± 14.9 vs. 70.3 ± 16.1, *p* < 0.05, g_Hedge_ = 0.42, [0.01–0.82]; Fig. 3). Gender and age, on the other hand, had no significant influence on the perceived overall workload (Fig. 3).

**Fig. 4:**
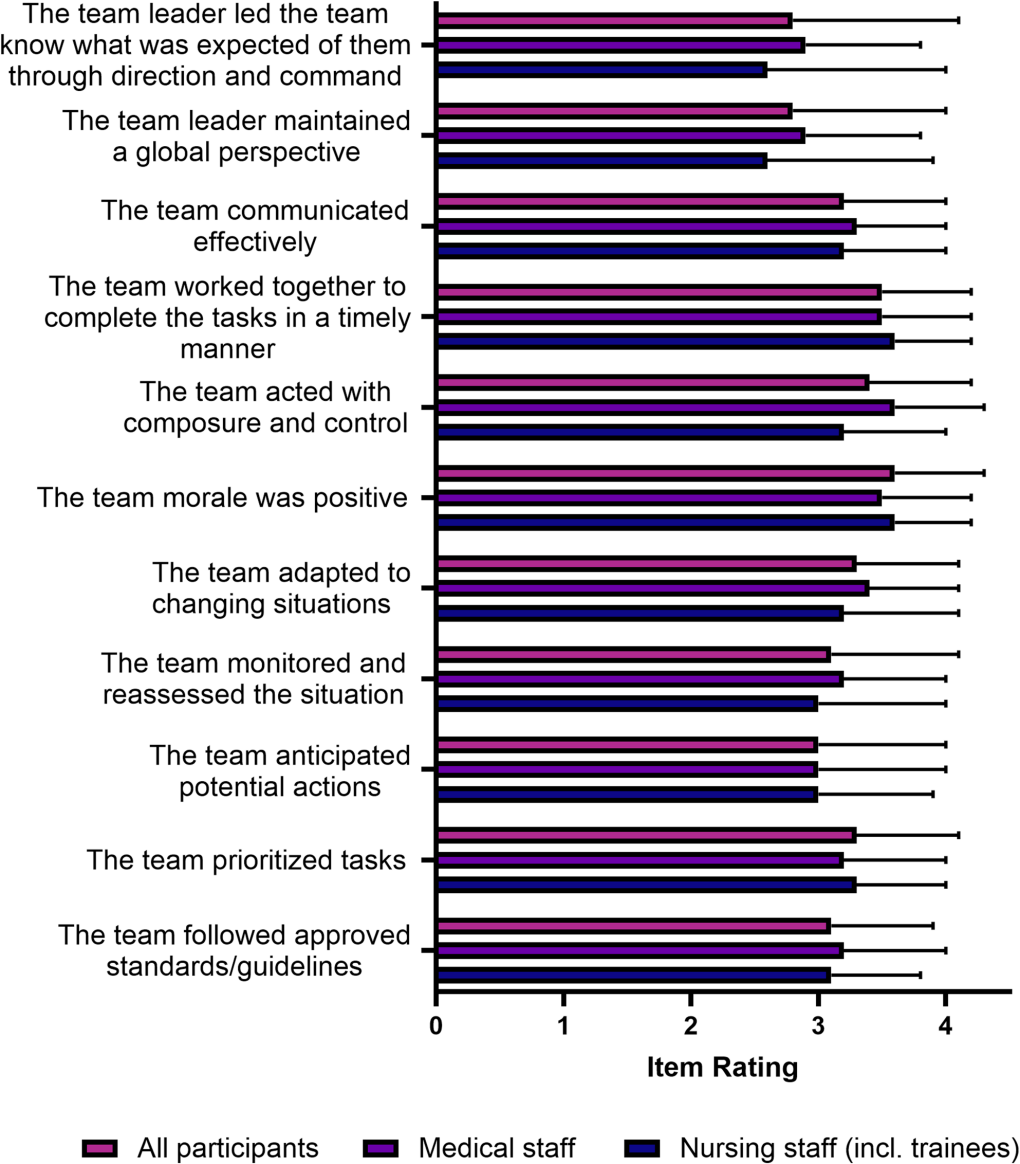
The Team Emergency Assessment Measure (TEAM) – Questionnaire. The eleven items (each from 0–4, higher values indicate better performance) of the TEAM questionnaire, divided into professional groups, Means (± SD) are shown. No significant differences were found when comparing the different groups

The results of the TEAM questionnaire show a relatively homogeneous picture in the assessment by the members of the individual professional groups. Regarding all participants the lowest scores were given to the two items relating to the team leader (2.8 ± 1.2 and 2.8 ± 1.3), while all other items were rated at an average of more than 3 out of 4 points. The items “*Team morale was positive”* (3.6 ± 0.7) and “*Team worked together to complete the tasks in a timely manner”* scored highest overall (3.5 ± 0.7; Fig. 4, Supplemental Table 2).

## Discussion

In context of pre-hospital MCI responses key performance indicators for evaluation have already been investigated [35–38]. However, there is no such definition for hospital MCI responses yet. There is limited evidence regarding effectiveness of MCI training for hospital staff. Several studies indicate that disaster drills can help train hospital staff effectively [39–41]. However, there is a need for scientific evaluation metrics of such training activities to better assess their true impact [21]. This study provides objectively measured benchmark data to enable evidence-based hospital emergency planning. The present study demonstrated that, in addition to the conventional observer-centered qualitative evaluation of MCI exercises, a methodology based on standardizable methods is also feasible. Different studies conducted in both preclinical and clinical settings have previously demonstrated the feasibility of GPS data loggers in accurately capturing patient trajectory data. These studies have been conducted in the context of an MCI simulation, and their findings have consistently shown that the use of GPS data loggers does not compromise the realism of the simulation [42, 43]. Conversely, the incorporation of additional uninvolved persons could potentially serve to enhance the realism of the exercise [42]. 

### Triage

Previous studies considered duration and accuracy for evaluation of triage systems [44]. Hospital-based MCI triage has a different approach with patient flow being a major concern. Triage, even when carried out by experienced trauma physicians, can be unreliable in an MCI [45]. Based on a full-scale simulation exercise, to our knowledge, this was the first time that the evaluation of screening quality and duration could be conducted entirely on the basis of data ascertained via geolocators, as evidenced by a full-scale simulation exercise. The screening duration and the accuracy of the process, including over- and undertriage, were both determined based on the expected triage category and the actual assignment to a category. In this MCI simulation exercise the proprietary triage algorithm of Heidelberg University Hospital was applied (Supplemental Fig. 1). In the literature, a hospital triage accuracy of 63.3% for single triage points and 70.0% for two-stage triage points has been reported [44]. In this study, a two-stage triage resulted in a triage accuracy of 75.4%. Category I (‘red’) patients could be identified significantly faster than other triage categories. The total triage time for patients classified as ‘red’, ‘yellow’, and ‘green’ is comparable to data from the literature. Carles et al. reported that mean triage time was 147 (± 105 SD) s in a real MCI response toward the terrorist attack in Nice in 2016 [46]. The reported data are based on 25 category I (‘red’) and 44 category II (‘yellow’) patients [46]. However, a significant proportion of undertriage was also observed (11.6% in category I). In comparison with the publication on the validation of the Berlin triage algorithm, on which the algorithm used is largely based, slightly better triage results were achieved [26]. The use of geolocators made it possible to identify falsely triaged case vignette. For instance, there was a recurrent undertriaging of case vignettes involving blast trauma with blood flow from the ear (*n* = 6 out of 7), such that this could be explicitly trained after the exercise and incorporated into the algorithm. Considering the undertriage among the other category I patients no systematic errors could be identified in the analysis of the patients in question and no corresponding evidence was found in the algorithm used. Based on the results, it cannot be assumed that these patients were triaged too quickly, as the triage times were not below average compared to those who were triaged correctly. It is important to consider other potential factors that may contribute to the observed phenomenon, including but not limited to spontaneous deviations from the established algorithm or the performance of the patient actors. This assumption is reinforced by the finding that the Berlin triage algorithm delivered superior results in a study with standardized case vignettes than could be demonstrated in full-scale exercises [27]. Further evaluation of this issue is recommended for future consideration, with the potential utilisation of geolocators as a useful instrument in this regard. The proportion of undertriage identified in this study is consistent with the findings of analyses conducted using other algorithms [47]. Another study by Vargas et al. [48] described the results of two triage groups. The initial accuracy was reported as 45.76% in the low experience group, 45.84% in the overtriage group, and 8.38% in the undertriage group. In the high experience group, the initial accuracy was 53.80%, overtriage 37.66%, and undertriage 8.57%. Subsequent training of both groups resulted in a significant enhancement of these results. [48] In this particular analysis, overtriage was found to be a negligible issue, with a rate of 2.9%. The results of the simulation can serve as a benchmark against which other hospitals can measure their own emergency exercises.

### Workload and perceived stress

In accordance with the findings of preceding studies, the NASA-TLX may be utilised as a metric to evaluate the pedagogical adequacy of scenarios, with the capacity to accurately reflect the stress levels experienced by participants [49]. In the present study, the overall workload of all participants was already in the upper stress level, with a mean score of 66.7 (± 16 SD). A meta-analysis of over 200 studies revealed that the 25th and 75th percentiles ranged from 39 to 61 in medical studies [50]. Interestingly, work experience and not profession or gender has been described to be related with increased self-reported perceived preparedness for MCIs [3]. Our data support this finding, by demonstrating that specialists and participants with a higher professional experience report less perceived overall workload in the NASA-TLX (Fig. 3). Another finding shows that although “frustration” was rated relatively low, it was higher among younger participants and resident physicians than among specialists (see Supplemental Table 1). These two findings demonstrate the efficacy of the NASA-TLX survey in identifying vulnerable groups within the staff population. It is recommended that these groups be given special attention in the preparation and follow-up of training and real-life scenarios. Another potential application of NASA-TLX is in full-scale simulations, with the purpose of providing a data set for the purpose of benchmarking the development and the evaluation of MCI training scenarios. Additionally, it could be used to monitor the effects of introducing new technologies or instruments in one’s own hospital during follow-up exercises [51, 52]. In a simulation study on MCI management, for example, a comparison of two groups showed that the group with artificial intelligence support had a significantly lower workload in the NASA-TLX score compared to the control group [52]. 

### Team performance

It has been demonstrated by preceding studies that non-technical skills are conducive to effective medical performance and can be enhanced through training [31–34, 53]. In this MCI simulation, interprofessional and interdisciplinary teams of three participants including a minimum of one physician were formed. The TEAM was thus employed as a reliable and validated instrument for the assessment of non-technical skills, with these skills being based on leadership qualities, teamwork, and task management [31, 33, 53]. The findings of the present study demonstrate that, in the face of a challenging environment and a considerable workload, the healthcare professionals exhibited above-average team performance, as evidenced by the NASA-TLX results. The mean TEAM item score was 3.2 (± 0.26) and the total TEAM score was 79.8%. A total of five simulation-based studies of professional teams reported a TEAM score ranging from 71.6% to 89.0%, with an average of 79% [32, 33, 53–55]. TEAM is a well-established instrument for the evaluation of emergency teams in acute situations, with documented improvements in scores following training sessions [32, 53, 56]. A survey of the literature reveals no precedent for the utilization of the TEAM questionnaire in MCI scenarios or simulations. Consequently, this study offers preliminary findings that can serve as a benchmark for future simulations and exercises in this domain.

A study published from Norway in 2025 suggests that many hospitals provide little education (15%) or training (26%) for major incidents involving a mass casualty situation [57]. It is reasonable to assume that the situation is similar in other regions. Publications from Germany suggest that exercises were carried out in between a quarter and a third of hospitals [58, 59]. The authors emphasize that it is crucial to back up each exercise with solid scientific research to gain the most valuable insights.

### Limitations

In this simulation, only adult trauma patients were simulated, which limits the generalizability of our findings to scenarios involving pediatric casualties or patients with other injury pattern. Pediatric patients require distinct triage algorithms and different care structures that are specifically tailored to the physiological and developmental needs of children. The participation in this exercise and the associated study was voluntary, which introduces potential biases that may affect the generalizability of the results. Participants who chose to take part may differ systematically from those who did not, possibly in terms of motivation, experience, or perceived competence in handling crisis situations. This selection bias could lead to an overestimation of the effectiveness of the training, as more motivated or experienced staff might perform better in a simulation setting. As the TEAM is primarily designed as an observer-rated tool, direct comparison is not possible. Thus, TEAM has been successful for its application as a tool for participants rating the non-technical skill perception of their respective team. While our findings provide valuable insights into the dynamics of patient flow and resource allocation, they should be interpreted with caution when applied to facilities with differing infrastructure. The simulation was stopped after arrival at an assigned operating theatre in case there was an indication for emergency surgery. Interpretation is therefore limited to MCI response prior to emergency surgery.

### Summary

The implications of our findings are significant for the evidence-based development of hospital emergency response plans. These insights can help other hospitals analyze their processes and identify potential inefficiencies that may exist regardless of the specific infrastructure. Furthermore, by offering a dataset that combines both objective and subjective metrics, this study strengthens the evidence base on hospital preparedness for MCIs, facilitating the comparability of exercises across institutions and over time when repeated. These findings can inform analyses of learning curves, highlight areas for improvement, and strengthen hospitals’ readiness for future emergency scenarios.

## Supplementary Information

Below is the link to the electronic supplementary material.

Supplementary Material 1

Supplementary Material 2

Supplementary Material 3

Supplementary Material 4

## Data Availability

Further information including geo-tracking video data is available in the supplement to this manuscript (Supplemental Video 1). Impressions of the entire exercise can also be gained from a publicly accessible video: https://www.youtube.com/watch?v=Pfu4D1Xh5CQ. Anonymized data are provided by the corresponding authors upon reasonable request.

## Declarations

### Patient and public involvement

Due to the nature and objective of this study, especially with health care staff acting as participants of this study, there was no involvement of patients or the public in design, conduct, reporting and dissemination of this study.
