# Supplementary figures and images for: Improving preparedness for mass casualty incidents in hospitals: insights from a large-scale simulation exercise with geotracking and validated questionnaires
Source: BMC Emerg Med. 2026 Mar 9;26:97. doi: 10.1186/s12873-026-01527-6 (PMC13064031; doi:10.1186/s12873-026-01527-6)

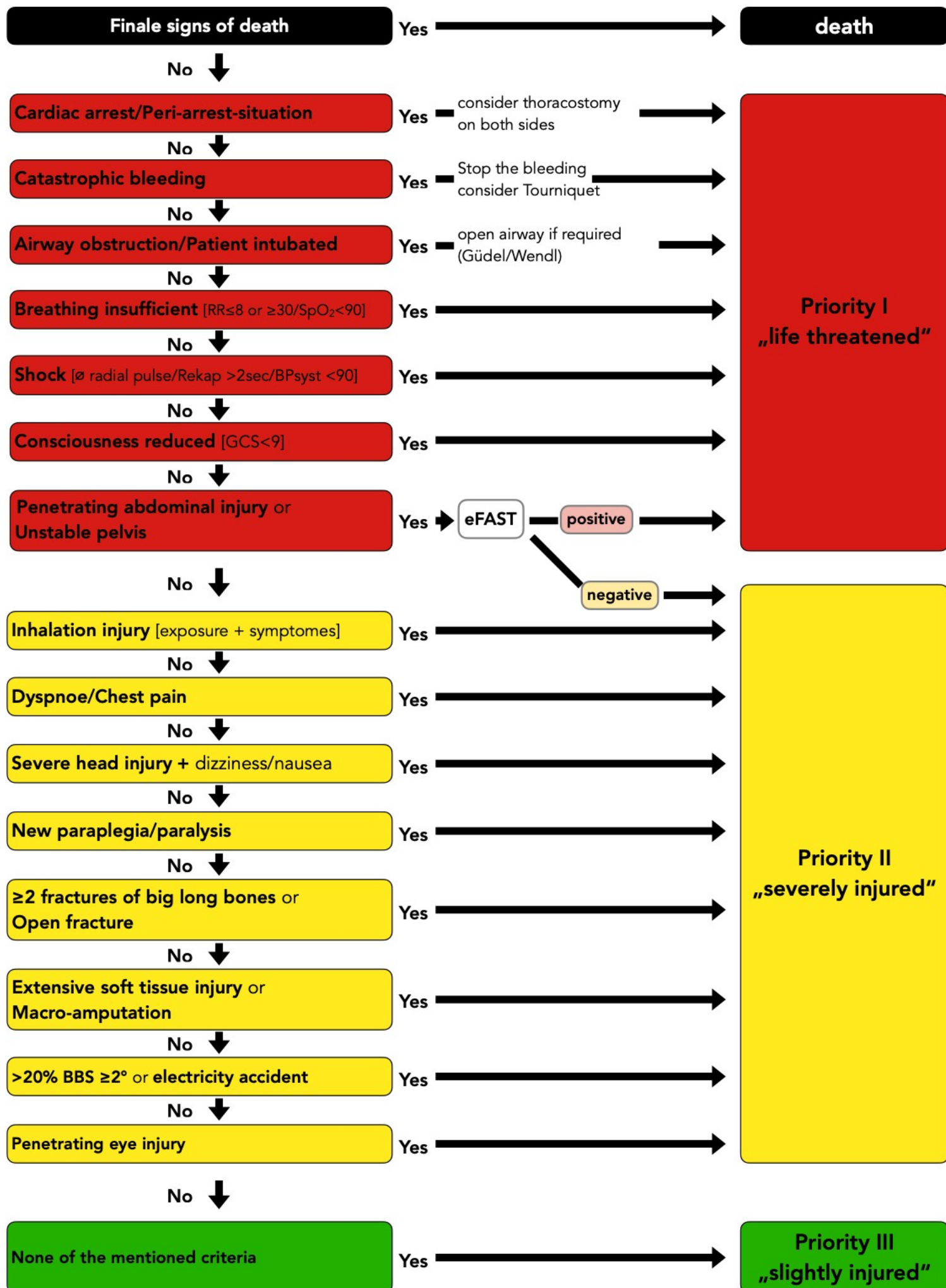

Supplement: Supplementary file 4 — Supplementary Material 4 [file 12873_2026_1527_MOESM4_ESM.pdf]
